# Ultrafines’ Quick Neurological Hit: Particles Take a Direct Route to the Brain

**Published:** 2006-08

**Authors:** Bob Weinhold

Proof of the penetrating capabilities of tiny particles continues to emerge. A team of U.S. researchers has just added to and clarified the existing evidence by documenting significant, rapid accumulations of inhaled ultrafine manganese oxide particles in the lung and many brain regions **[*EHP* 114:1172–1178; Elder et al.]**. They also demonstrated that particles don’t need to dissolve to spread, and that inhalation pathways can be more efficient than circulatory ones.

The researchers evaluated the translocation and tissue distribution of manganese oxide ultrafines in rats that had inhaled a nearly insoluble form of these solid particles for six hours per day, at a concentration in the mid-range typically experienced by welders. After 12 days of exposure, the manganese concentration in the olfactory bulb (a region of the brain that abuts the nasal cavity) had increased about 3.5-fold. At the same time, lung manganese concentrations doubled, and there were small but significant increases in other brain regions, such as the cerebellum, the frontal cortex, and the striatum.

The inhaled ultrafines didn’t cause obvious lung inflammation. However, in the brain several markers of inflammation and stress response, including tumor necrosis factor and macrophage inflammatory protein, increased by anywhere from about 2- to 30-fold.

To determine how inhaled manganese oxide ultrafines spread, the team closed the right nostril of several of the rats and had them inhale manganese oxide solely through the left nostril. They found that the vast majority of manganese quickly accumulated in the left olfactory bulb. This suggested that very little of the accumulation was due to other routes, such as dissolution and distribution via the circulatory system; otherwise, the manganese would have appeared in both olfactory bulbs.

The negligible role of the circulatory system contrasted with the findings of another manganese study, but that study utilized poorly soluble manganese phosphate particles that were several orders of magnitude larger than the approximately 30-nm manganese oxide agglomerates used here. The particles in the current study were about one-sixth the diameter of the olfactory neurons, along which the agglomerates moved into the brain.

These findings, as well as those of other studies of tiny particles such as carbon, gold, poliovirus, and engineered nanoparticles, suggest to the researchers that much more research is needed to determine if other inhaled ultrafines can also rapidly disseminate and cause effects throughout animal bodies.

